# Neuroplasticity of Speech-in-Noise Processing in Older Adults Assessed by Functional Near-Infrared Spectroscopy (fNIRS)

**DOI:** 10.1007/s10548-024-01070-2

**Published:** 2024-07-23

**Authors:** Guangting Mai, Zhizhao Jiang, Xinran Wang, Ilias Tachtsidis, Peter Howell

**Affiliations:** 1 National Institute for Health and Care Research Nottingham Biomedical Research Centre, Nottingham, UK; 2 Academic Unit of Mental Health and Clinical Neurosciences, School of Medicine, University of Nottingham, Nottingham, UK; 3 Division of Psychology and Language Sciences, University College London, London, UK; 4 Department of Medical Physics and Biomedical Engineering, University College London, London, UK; 5 Max Planck Institute for Human Cognitive and Brain Sciences, Leipzig, Germany

**Keywords:** Functional near-infrared spectroscopy (fNIRS), Auditory neuroplasticity, Older adults, Speech-in-noise perception

## Abstract

Functional near-infrared spectroscopy (fNIRS), a non-invasive optical neuroimaging technique that is portable and acoustically silent, has become a promising tool for evaluating auditory brain functions in hearing-vulnerable individuals. This study, for the first time, used fNIRS to evaluate neuroplasticity of speech-in-noise processing in older adults. Ten older adults, most of whom had moderate-to-mild hearing loss, participated in a 4-week speech-in-noise training. Their speech-in-noise performances and fNIRS brain responses to speech (auditory sentences in noise), non-speech (spectrally-rotated speech in noise) and visual (flashing chequerboards) stimuli were evaluated pre- (T0) and post-training (immediately after training, T1; and after a 4-week retention, T2). Behaviourally, speech-in-noise performances were improved after retention (T2 vs. T0) but not immediately after training (T1 vs. T0). Neurally, we intriguingly found brain responses to speech vs. non-speech decreased significantly in the left auditory cortex after retention (T2 vs. T0 and T2 vs. T1) for which we interpret as suppressed processing of background noise during speech listening alongside the significant behavioural improvements. Meanwhile, functional connectivity within and between multiple regions of temporal, parietal and frontal lobes was significantly enhanced in the speech condition after retention (T2 vs. T0). We also found neural changes before the emergence of significant behavioural improvements. Compared to pre-training, responses to speech vs. non-speech in the left frontal/prefrontal cortex were decreased significantly both immediately after training (T1 vs. T0) and retention (T2 vs. T0), reflecting possible alleviation of listening efforts. Finally, connectivity was significantly decreased between auditory and higher-level non-auditory (parietal and frontal) cortices in response to visual stimuli immediately after training (T1 vs. T0), indicating decreased cross-modal takeover of speech-related regions during visual processing. The results thus showed that neuroplasticity can be observed not only at the same time with, but also *before*, behavioural changes in speech-in-noise perception. To our knowledge, this is the first fNIRS study to evaluate speech-based auditory neuroplasticity in older adults. It thus provides important implications for current research by illustrating the promises of detecting neuroplasticity using fNIRS in hearing-vulnerable individuals.

**Supplementary Information:**

The online version contains supplementary material available at 10.1007/s10548-024-01070-2.

## Introduction

How the brain processes speech is an important topic in auditory cognitive neuroscience. A long-standing focus is to study the brain functions in hearing-vulnerable populations such as older adults and hearing-impaired listeners who experience challenges in speech and language perception (see reviews: Peelle and Wingfield 2016; Slade et al. 2020). This current study asks questions on how contemporary sophisticated functional neuroimaging techniques can help us practically study this essential topic. Over the years, studies have used techniques, such as functional magnetic resonance imaging (fMRI) and positron emission tomography (PET), to illustrate the breakdown of brain processing of speech and language in older and hearing-impaired listeners (Wong et al. 1999, 2009; Peelle et al. 2011; Vaden et al. 2015, 2016; Vogelzang et al. 2021). Both fMRI and PET detects dynamics of cerebral haemoglobin (haemodynamic responses) at different regions of the brain capturing neural responses with high special resolution and has been widely used in auditory research (e.g., Zatorre et al. 1996; Zatorre 2001; Hall et al. 1999, 2009; Peelle 2014). Using these techniques, studies have observed altered neural sensitivity to speech signals in the auditory cortices (Wong et al. 1999, 2009; Peelle et al. 2011) as well as abnormal neural responses at higher-level non-auditory, cognitive regions in these individuals compared to normal-hearing young adult listeners (Wong et al. 1999, 2009; Vaden et al. 2015, 2016; Vogelzang et al. 2021). While both fMRI and PET have been widely used, they also face limitations in auditory research. For example, both techniques can be expensive and may not be always easy to use for large-scale studies in clinical populations (Boas et al. 2014; Pinti et al. 2020). Also, fMRI generates loud extraneous scanning noise that can cause problems for assessing auditory functions (Scarff et al. 2004; Gaab et al. 2007). Furthermore, hearing protheses, like hearing aids and cochlear implants, can have intensive magnetic interference with MRI scanning (Saliba et al. 2016; Basura et al. 2018; Harrison et al. 2021) such that hearing aid and cochlear implant users are largely excluded from fMRI research. For PET, although it is noise-free and does not have magnetic interactions with hearing protheses, it requires an invasive procedure, i.e., injection of radioactive isotopes (Johnsrude et al. 2002), making it unsuitable for repetitive use in clinical populations. Besides fMRI and PET, functional near-infrared spectroscopy (fNIRS) is another promising technique to study the neural processes of auditory and speech perception (Pollonini et al. 2014; Wiggins et al. 2016; Defenderfer et al. 2017, 2021; Wijayasiri et al. 2017; Lawrence et al. 2018; Mushtaq et al. 2021; Zhou et al. 2022). fNIRS is an optical imaging technique that illuminates scalp of the brain using near-infrared light and measures the intensity of light returning from cortical areas through which haemodynamic responses are estimated (Boas et al. 2014; Pinti et al. 2020). Besides neural activity in the cortical areas, these fNIRS-detected haemodynamic responses could also capture non-neural, systemic physiological confounders in the extracerebral layers, including changes in heart-beat, respiration, blood pressure and autonomic nervous system activities (see a review by Tachtsidis and Scholkmann 2016). However, after applying appropriate signal processing to appropriately attenuate these confounds (e.g., adaptive filtering, regressing out/subtracting physiological measurements etc., see Tachtsidis and Scholkmann 2016), it is evident that fNIRS signals well represent cortical neural activities particularly in auditory experiments. For example, Chen et al. (2015) tested combined fNIRS and electroencephalographic (EEG) responses to low-level auditory (amplitude-modulated tones) and visual (flashing chequerboards) stimuli in healthy normal-hearing human participants. They found that fNIRS responses are most robust to stimuli in sensory regions of the corresponding modalities (i.e., strongest responses to auditory stimuli in the auditory cortex and to visual stimuli in the visual cortex, respectively) and that the strengths of fNIRS responses in the auditory cortex are significantly correlated with the corresponding auditory-evoked EEG activities. Another example is that fNIRS activities can index intelligibility (in the auditory cortex) and higher-level cognitive functions (e.g., listening effort in the left frontal cortex) in response to complex acoustic stimuli such as speech in normal-hearing participants (Lawrence et al. 2018). Also, auditory cortical fNIRS responses to speech are shown to have significant test-retest reliability after applying appropriate physiological attenuation techniques (Wiggins et al. 2016).

Nowadays fNIRS has become more advantageous and practical to use in hearing-vulnerable populations to study their auditory brain functions (Boas et al. 2014; Pinti et al. 2020). Compared to fMRI or PET, fNIRS is more portable and relatively less expensive, hence easier to use in laboratory environments for clinical populations (Boas et al. 2014; Pinti et al. 2020). Also, compared to fMRI, fNIRS is acoustically silent which is crucial for auditory experiments in those who face challenges in hearing and speech. Furthermore, unlike PET, fNIRS is non-invasive, making it more suitable for repeated measurements, e.g., in longitudinal studies, for clinical populations (Saliba et al. 2016; Basura et al. 2018; Harrison et al. 2021). Lastly, fNIRS is compatible with people who wear hearing protheses like hearing aids and cochlear implants which can have intensive magnetic interference with MRI scanning (Saliba et al. 2016; Basura et al. 2018; Harrison et al. 2021). Besides studies in normal-hearing participants as mentioned above, recent research has successfully used fNIRS to illustrate the neural processes of hearing and speech perception in hearing-vulnerable populations. For instance, using fNIRS, Olds et al. (2016) showed that cochlear implant listeners with good speech perception exhibited greater auditory cortical activations in response to intelligible than unintelligible speech whilst those with poor perception did not show distinguishable activations, revealing the association between speech perception and auditory cortical activities in these individuals. Previous studies have also shown successes in detecting listening efforts using fNIRS in older and hearing-impaired listeners. Rovetti et al. (2019) showed that reduction of fNIRS prefrontal cortical activations (reflecting alleviation in listening effort) during an auditory N-back task is associated with the use of hearing aids in older adults with hearing loss. Sherafati et al. (2022) showed greater fNIRS prefrontal cortical activations in cochlear implant listeners than normal-hearing controls during spoken word listening tasks, reflecting greater listening efforts in the implanted listeners. fNIRS also demonstrated promises in detecting cross-modal activations in relation to speech perception in the hearing-impaired. For instance, Anderson et al. (2017) showed that better speech perception in cochlear implant listeners is associated with enhanced fNIRS cross-modal activations (auditory cortical responses to visual speech). Fullerton et al. (2022) further showed better speech perception is associated with functional connectivity between auditory and visual cortices in response to visual speech in implanted listeners.

Despite these successes of the use of fNIRS and its unique advantages, previous research also confronted limitations of this technique. For example, compared to neuroelectromagnetic methods like electroencephalography (EEG) and magnetoencephalography (MEG), fNIRS measures haemodynamic responses that are sluggish, so it is unable to capture fine-grained timing information of the neural signals (Pinti et al. 2020). Also, its restricted depth of optode penetration makes it only detects neural activations occurred in the outer cortices with a relatively sparse spatial resolution compared to fMRI and PET which can further capture activities within sulci and deep into medial cortices (Pinti et al. 2020). Hence, it is worth noticing these limitations due to which some brain functions may not be easily detected through fNIRS. Therefore, evaluating the feasibility of this technique as discussed above is an important step to confirm its promises in auditory research. However, most of these efforts so far have focused on cross-sectional experiments and it is unclear how *changes* in brain functions over time could be feasibly detected by fNIRS. Such changes are referred as ‘neuroplasticity’, which reflects the capacity of the brain to undergo functional reorganization across time (Cramer et al. 2011). Literature that assessed auditory neuroplasticity using fNIRS is scarce. Anderson et al. (2017) has documented changes in auditory cortical responses to visual speech (cross-modal neuroplasticity) over time in severe hearing-impaired individuals like cochlear implant listeners, showing that enhancement in such responses is associated with better auditory speech perception. However, it is unclear whether these changes are susceptible to auditory stimuli (as opposed to visual stimuli used in Anderson et al. 2017) and whether neuroplasticity also undergoes in other brain regions besides the auditory cortex. More importantly, it is not clear whether this can be observed in more typical individuals who have less impaired hearing than cochlear implant listeners in the first place, e.g., in those who experience normal ageing that is often accompanied by mild-to-moderate sensorineural hearing loss (Gopinath et al. 2009; Humes et al. 2010). Observing this plasticity is important because it should pave the way for future research into the neural mechanisms underlying the behavioural changes, especially in older adults and hearing-vulnerable populations who have shown the potential to improve their speech perception after proper speech-based training interventions (Stropahl et al. 2020; Bieber and Gordon-Salant 2021). Clinically, it can help identify those who have strong potentials for positive neuroplastic changes so that individualized treatments can be properly designed (Cramer et al. 2011; Nahum et al. 2013).

In the current study, we aim to test the hypothesis that fNIRS is able to detect auditory neuroplasticity in older adults when they perceive speech perception in noisy environments. We predict that the plastic changes can be induced and observed by fNIRS after appropriate speech-based training over time. These should include cortical changes in activations and functional connectivity between auditory and higher-level cognitive regions in response to speech stimuli, as well as changes in auditory cortical activations in response to stimuli of a non-auditory modality (e.g., visual) according to changes in cross-modal maladaptation. To this end, we conducted a longitudinal experiment in older adults, most of whom had mild-to-moderate sensorineural hearing loss. Participants received a four-week home-based speech-in-noise training and their brain activities were measured by fNIRS over the speech- and language-related cortical areas (temporal, parietal and frontal regions, see Poeppel and Hickock, 2007) both before and after training. Previous evidence has shown that such training leads to improved behavioural performances in those with hearing disorders. For example, the same training program that we used here was found to significantly improve speech-in-noise perception in cochlear implant listeners (Green et al. 2019). There has also been evidence showing that similar training results in improved speech-in-noise performances in older adults with mild-to-moderate hearing loss (e.g., Sweetow and Sabes 2006; Lai et al. 2023). Here, besides assessing behavioural changes, neural responses at various brain regions of interest as well as functional connectivity were examined during an auditory and a visual test and were compared between sessions before and after training. In the auditory test, participants listened to speech (spoken sentences) and non-speech stimuli (spectrally-rotated versions of speech) presented in noisy backgrounds. Spectrally-rotated speech preserves acoustic properties similar to original speech, including amplitude modulations, harmonic complexity and intonations over time, but was highly unintelligible (Scott et al. 2000, 2009). Comparing speech with spectrally-rotated speech hence controlled for the acoustic complexity to study how neuroplasticity may be related to speech-specific factors such as intelligibility. We expect increased auditory cortical activities reflecting greater auditory sensitivity after training as well as decreased left frontal/prefrontal cortical activities reflecting reduced listening efforts (Wild et al. 2012; Wijayasiri et al. 2017; Rovetti et al. 2019; Sherafati et al. 2022). We also expect enhancements in brain connectivity reflecting better coordination between language-related areas (Poeppel and Hickock, 2007). In the visual test, participants were exposed to speech-unrelated visual stimuli (flashing chequerboards). Previous research has reported that such stimuli can elicit greater auditory cortical activities in hearing-impaired people reflecting cross-modal maladaptation associated with poorer speech perception (Campbell and Sharma 2014; Chen et al., 2016; Corina et al. 2017). We expect that this maladaptation would be reduced after training (i.e., reduced auditory cortical activities and/or reduced connectivity between auditory cortex and higher-order parietal and frontal speech-related areas in response to visual stimuli). We anticipate that the observed longitudinal changes should provide new insights into possible underlying mechanisms for changes in speech-in-noise perception over time.

## Methods and Materials

This study was approved by the UCL Research Ethics Committee. All participants were consent and reimbursed for their participation.

### Participants

**Fig. 1:**
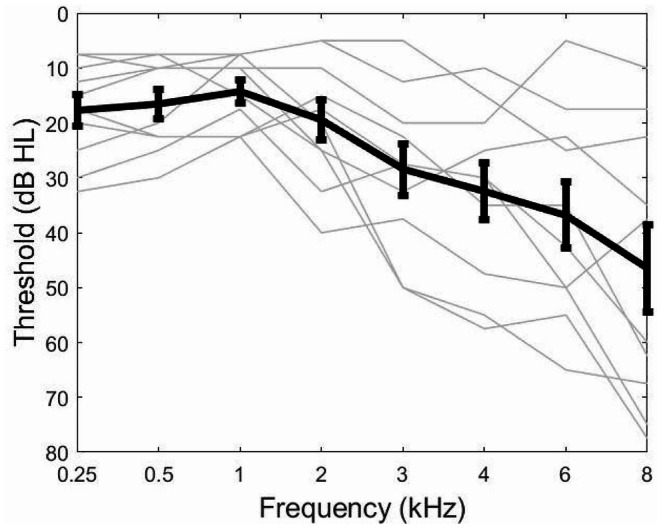
Audiograms of participants averaged across the two ears. Grey lines show the thresholds of individual participants and the black line show the thresholds averaged across participants. Error bars indicate the standard errors of the means

Ten right-handed, healthy adult participants (two males) aged between 63 and 78 years (mean = 70, SD = 4.5) were recruited. They were all native British English speakers with no reported histories of neurological, cognitive (e.g., Mild Cognitive Impairment) or language disorders. Their pure-tone audiograms (PTAs) were measured for each ear before the speech-in-noise training using a MAICO MA41 Audiometer (MAICO Diagnostics, Germany) at 0.25, 0.5, 1, 2, 3, 4, 6 and 8 kHz. Two participants had normal hearing (≤ 25 dB HL) at all frequencies ≤ 6 kHz in both ears. The other eight showed a general pattern of mild-to-moderate hearing loss (30–60 dB HL) especially at high frequencies (> 2 kHz) (see Fig. 1). This therefore matches the real-life scenario where majority of healthy ageing populations suffer from high-frequency mild-to-moderate hearing loss (Gopinath et al. 2009; Humes et al. 2010). In addition, according to self-report, no participants ever used hearing aids before the current study.

### Design

Participants received a home-based speech-in-noise training through a participant-/patient-friendly App developed by Green et al. (2019). With proper instructions, participants were able to complete the training process by themselves via controlling the Matlab Graphical User Interfaces using a computer tablet at their own homes. Training data were saved in an online UCL Research Dropbox in a daily basis so that experimenters could make sure the training was gone through smoothly. During the training, participants listened to storybooks (in British English) spoken by a male and a female speaker sentence-by-sentence presented in background noise and they were asked to identify words within each sentence through multiple-choice word tasks. The background noises were multiple-talker babbles (4, 8 and 16 talker-babbles presented throughout the training in intermixed orders; half males and half females). An adaptive procedure was adopted where the signal-to-noise ratio (SNR) increased/decreased following the decreases/increases in participants’ accuracies over time to keep their attention. The training lasted for 4 weeks, 6 days per week, ~ 30 min per day.

**Fig. 2:**
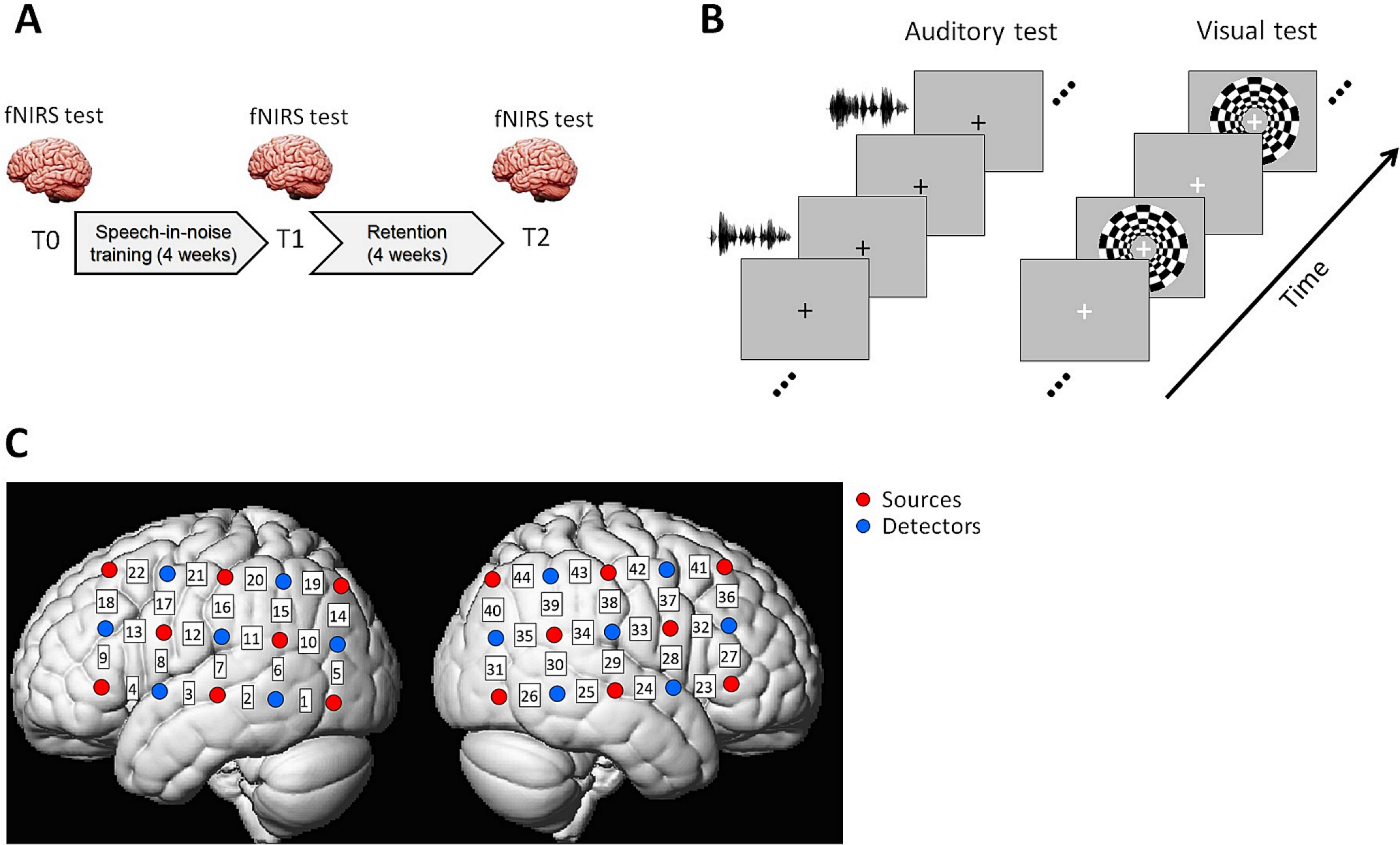
Experiment design. **(A)** Participants completed a 4-week home-based speech-in-noise training and their brain functions were measured by fNIRS before (T0) and after the training (T1 and T2). **(B)** The fNIRS experiment included an auditory test where participants listened to auditory sentences (speech and spectrally rotated speech) and a visual test where participants watched a flashing chequerboard. A block design was adopted with resting blocks interleaved between the auditory/visual stimuli. **(C)** Optode configuration of the fNIRS experiment was two 5-by-3 probe sets that formed 44 channels (22 channels in each hemisphere) over speech- and language-related temporal, parietal and frontal cortical regions (left: left hemisphere; right: right hemisphere). Red and blue circles denote the sources and detectors, respectively. The channel indices are indicated in the white squares between the sources and detectors

Their speech-in-noise performances and brain responses to auditory and visual stimuli were measured both before (a day or two before the training as the baseline, T0) and after training (the next day after the training ended, T1; and after an additional 4-week retention period, T2). Figure 2A illustrates the study procedure.

### Speech-in-Noise Tasks

The speech-in-noise performances were measured as participants’ speech reception thresholds (SRT) when they listened to short sentences in noisy backgrounds. The sentences were chosen from the Adaptive Sentence List (ASL), each of which consists of three key (content) words (e.g., ‘He wiped the table’ with key words ‘he’, ‘wiped’ and ‘table’) spoken by a male native British English speaker (MacLeod and Summerfield 1990). Participants were seated in a quiet room listening to 30 sentences under an 8-talker babble noise (the same 8-talker babbles as in the training) via inserted earphones (ER-3 insert earphone, Intelligent Hearing Systems, USA). They were required to verbally report as many words as they could for each sentence. The signal-to-noise ratio (SNR) was initially set at 6 dB for the first sentence (for which all participants were able to recognize all key words) and was decreased by 4 dB for subsequent sentences until < 50% words (i.e., < 2 words) were correctly reported. SNR was then decreased by 2 dB when word correctness was greater than 50% (i.e., participants reported back two or three out of three key words), otherwise SNR was increased by 2 dB, for each of the following sentences. The SRT was measured as the mean SNR across all reversals at the step size of 2 dB (Schoof and Rosen 2014). The number of reversals was between 15 and 19 depending on participants’ actual performances. Therefore, lower SRT reflects better speech-in-noise performance. The overall sound level (sentence plus noise) was calibrated and fixed at 75 dB SL. The procedure was controlled using Matlab 2016a (Mathworks, USA) with key words for each sentence appearing on the computer screen seen only by the experimenters. The ‘loose keyword scoring’ approach was followed, meaning that a reported word was considered correct as long as it matched the root of a key word (e.g., ‘stand’ was considered correct for the keyword ‘stood’) (Macleod and Summerfield 1990). There were 6 practice sentences prior to each formal test. Contents of the testing sentences all differed across different testing sessions (T0, T1 and T2).

### fNIRS Experiments

#### Optode Placements

Brain haemodynamic responses were recorded by a continuous-wave fNIRS system (ETG-4000, Hitachi Medical, Japan; sample rate of 10 Hz) that uses two wavelengths of light at 695 and 830 nm to allow the estimates of changes in both oxy- (HbO) and deoxy-haemoglobin (HbR). The haemodynamics were measured using two 5-by-3 optode probe sets (8 sources and 7 detectors with a fixed adjacent source-detector distance of 3 cm on both hemispheres), hence 44 channels covering much of the temporal, parietal and frontal areas (see Fig. 2C). These areas are consistent with the some of the most important cortical regions that contribute to human processing of speech and language (Hickok and Poeppel 2007). To ensure that the channels are in largely the same positions across participants, the probe sets were fitted on a specific cap based on the international 10–20 system (channel 7/29 corresponds to T7/T8 near the left/right auditory cortex). All participants wore the same cap. The vertex position and the nasion-vertex-inion midline were aligned across participants. To fit the channel positions on the cortical anatomy, the optodes and anatomical surface landmarks (nasion, vertex, inion, left and right ears) were registered using a 3D digitizer provided by the EGT-4000. In practice, it had shown difficult to appropriately register the landmarks in many of our participants (e.g., very small dislocations of digital sensors can cause greatly spurious head shape). Therefore, we used the most successful digitization result in one of the participants as the representative for channel positioning over the anatomical areas for all participants. All participants’ head sizes fell within 54–58 cm for the head circumference and 33–35 cm for the ear-to-ear measurement over the vertex, indicating that interindividual differences were no greater than 10% (in cm). Also, while the 10–20 system located the auditory cortices (channels 7 and 29 in the left and right hemispheres, respectively), channels that were most distant from auditory cortices were channels 14, 18 (left hemisphere), 36 and 40 (right hemisphere) (see Fig. 2C) which were ~ 6.7 cm away given the adjacent source-detector distance at 3 cm. Therefore, less than 10% individual variability in head size indicate that any possible fNIRS channel location inconsistency was 6–7 mm at maximum (10% of 6.7 cm is 6–7 mm), assuming that all participants were fitted with a common MNI atlas. Such scale is less than a quarter of the 3-cm source-detector distance. Because of the relatively sparse spatial resolution of fNIRS compared to techniques like fMRI, we suggest that such maximal possible inconsistency is largely acceptable in practice, particularly for the results based on signal averages across channels in specific regions of interest (see *fNIRS data analyses* for details) which further attenuate negative effects due to possible dislocation of single channels. Therefore, we argue that the standardized alignment procedure currently followed should *not* lead to large interindividual variability of channel positions with pronounced effects on the neural measurements.

Efforts were taken by the experimenters to maximize the good optode contacts with the scalp. With participants who had hair, a thin stick was used to help pull out the hair out of the way between the optodes and the scalp. General good contacts were ensured with waveforms having clear cardiac elements monitored by ETG-4000 in real-time. Formal tests started when better contacts could no longer be achieved after every effort was taken. Channels with poor signal quality were further detected and excluded for subsequent analyses (see *fNIRS data analyses*).

#### Paradigms

The fNIRS experiments included an auditory and a visual test. The auditory test used speech and non-speech stimuli. The speech stimuli were ASL sentences spoken by the same male speaker as in the speech-in-noise tasks while non-speech stimuli were spectrally-rotated versions of the speech (Scott et al. 2000, 2009). The spectrally-rotated speech preserves some of the acoustic properties of the original speech, including similar wideband amplitude modulations, harmonic complexity and intonations, but was highly unintelligible (Scott et al. 2000, 2009). This thus controlled for the auditory processing of acoustic properties that enabled us to study how neuroplasticity may be related to speech-specific factors such as intelligibility. All stimuli were presented via ER-3 earphones under an 8-talker babble noise with the overall sound level (sentence plus noise) calibrated at 75 dB as in the speech-in-noise tasks. The SNR was fixed across all testing sessions at the SRT obtained from the speech-in-noise task at T0 on a participant-by-participant basis (the average SNR across participants was −1.17 dB). This ensured that speech stimuli were partly intelligible (~ 50% word recognition at T0) which thus required similar listening efforts across participants and that neural responses to the speech/non-speech stimuli can be statistically compared across different sessions. Contents of the sentences were all different from the behavioural speech-in-noise tasks and differed across sessions.

A block design was adopted in which participants sat in front of a computer screen with a grey background and a black cross in the middle for them to keep their eyes on and listened to 12 speech and 12 non-speech blocks presented in a randomized order (see Fig. 2B). Each block consisted of 4 sentence trials. All sentences were ~ 2 s long and each sentence plus noise was set to a fixed duration of 2.5 s that allowed the babble noise to start before sentence onset and extend after the sentence ended. Another 2.5 s’ silent interval followed each sentence before the next during which participants were required to gently press a button (1, 2 or 3) to indicate how many key words they could recognize from the sentence. Each block thus lasted 20 s. Silent resting blocks were interleaved between the speech and non-speech blocks, each of which had a duration set randomly at 15, 17, 19 or 21 s. This was to reduce the possibility of participants being able to predict when the next speech/non-speech block would happen. The auditory test lasted for ~ 15 min.

For the visual test, participants were exposed to a flashing radial chequerboard with black and white patches (the two colours reversed at the rate of 8 Hz, see Vafaee and Gjedde 2000) on the computer screen against a grey background. Similar to the auditory test, a block design was used (see Fig. 2B). There were 10 chequerboard blocks, each with a duration of 20 s. In addition to the chequerboard, a white cross appeared in the middle of the screen and was set to change to red and then back to white (once for each change during each block; timings for the changes were set at random but occurred no earlier than 4 s after the block onset). To ensure participants’ engagement, they were asked to focus on the cross and gently press a button whenever the colour changed. Resting blocks were interleaved between stimulus blocks, each with a duration randomly set at 17, 20, or 23 s. The visual test lasted for ~ 7 min.

A two to three minutes’ practice run was provided before formally starting each test so that participants were familiarized with the paradigms. Across the entire test period, participants were asked to restrain their body and head movements and consistently keep their eyes on the cross in the middle of the screen.

**Fig. 3:**
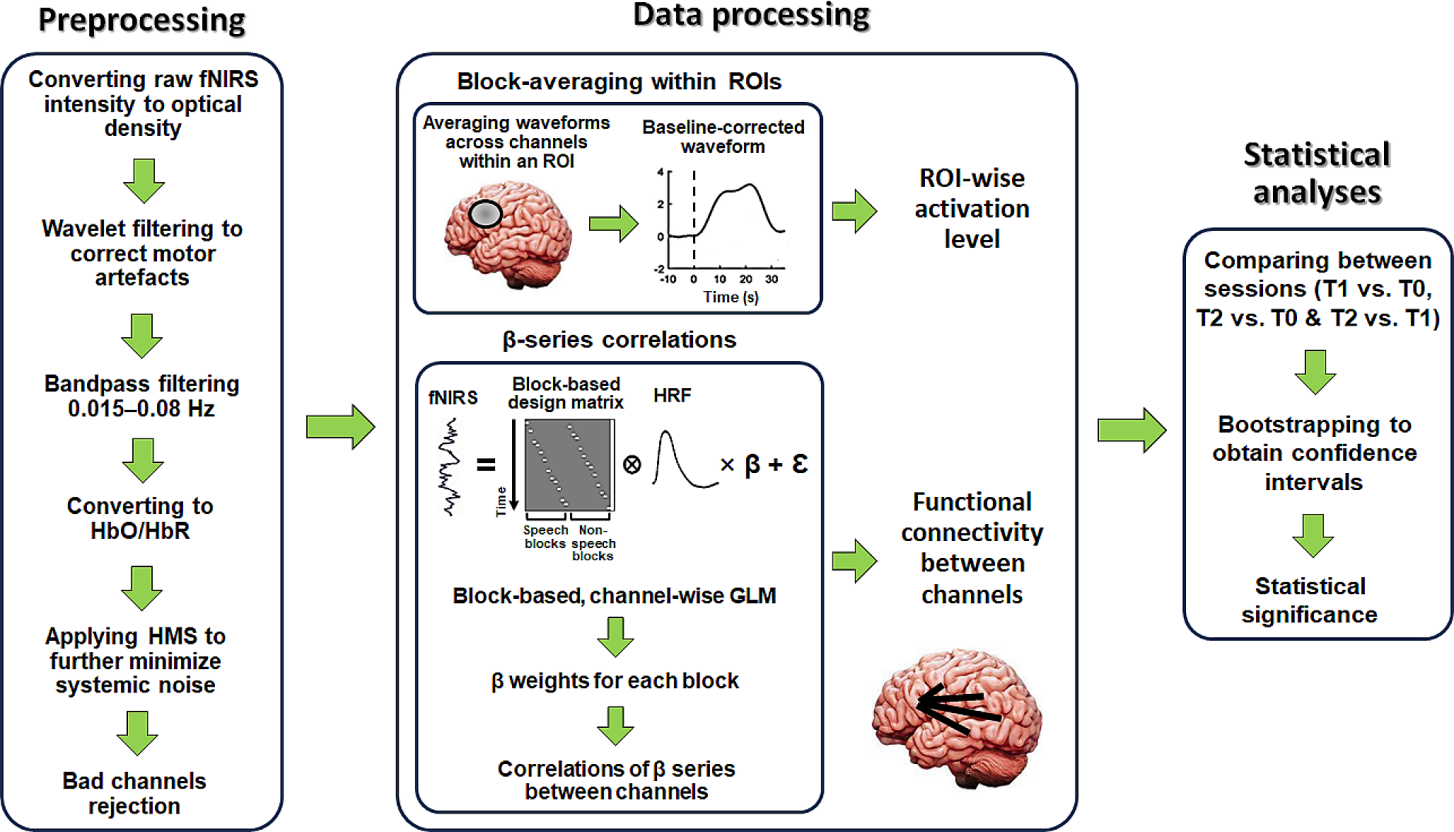
Flow charts for signal processing. The raw fNIRS data were first preprocessed. This included conversion fNIRS intensity to optical density, motor artefact correction (via wavelet filtering), bandpass filtering, conversion to HbO and HbR, and applying haemodynamic modality separation (HMS). Bad channels were finally detected via scalp coupling index (SCI) and were rejected for subsequent analyses. The preprocessed data were then used to measure functional activation levels and connectivity for each task (auditory and visual) during each session (T0, T1 and T2). Activation levels were measured via normalised response magnitudes by block-averaging within ROIs. Functional connectivity was measured by correlations of block-based beta-weight (through GLM) series between individual channels. Statistics were finally conducted using bootstrapping to obtain confidence intervals based on comparisons of activation levels and connectivity between different sessions for each task. Details of the entire procedure are described in the main text

### fNIRS Data Analyses

The signal processing procedure includes preprocessing, data processing of functional activations and connectivity, and statistical analyses. Figure 3 shows the flow charts of this procedure.

#### Preprocessing

All signal processing and analyses of fNIRS were conducted using Matlab 2019b (Mathworks, USA) combining customized codes, and the HOMER2 (Huppert et al. 2009) (homer-fnirs.org) and SPM-fNIRS toolbox (Tak et al. 2016) (www.nitrc.org/projects/spm_fnirs). We followed the signal processing procedure which was reported to result in high test-retest reliability of speech-evoked responses by fNIRS (Wiggins et al. 2016).

The raw fNIRS intensity signals were first converted to changes in optical density (via the HOMER2 function *hmrIntensity2OD*). Then motion artefacts were corrected using wavelet filtering (via the HOMER2 function *hmrMotionCorrectWavelet*). This removed wavelet coefficients lying more than 0.719 times the inter-quantile range below the first or above the third quartiles (Lawrence et al. 2018; Mushtaq et al. 2021). The optical density signals were then bandpass filtered between 0.015 and 0.08 Hz using a zero-phase 3rd-order Butterworth filter (hence covering the presentation frequency of ~ 0.025 Hz in the block design) which attenuated the low-frequency drifts and changes in arterial blood pressure, respiratory and cardiac activities. The signals were then converted to changes in HbO and HbR concentrations via the modified Beer-Lambert Law (Huppert et al. 2009). The haemodynamic modality separation (HMS) algorithm (Yamada et al. 2012) was finally applied to further minimize the possible remaining systemic physiological noise and motion artefacts (e.g., slow head and body motions) (Wiggins et al. 2016). While temporal smoothing methods such as pre-whitening and pre-colouring are suggested to further attenuate physiological noise by removing temporal autocorrelations (Yücel et al. 2021), they lead to further low/high-pass filtering of the signals. As the bandwidth of the current preprocessed fNIRS was sufficiently narrow (0.015–0.08 Hz), further smoothing may wipe out useful neural signals. We thus applied HMS that has been proved effective in fNIRS (Wiggins et al. 2016) instead of pre-whitening/pre-colouring. HMS is based on the fact that changes in HbO and HbR are negatively correlated in the functional responses but positively correlated in the motion and physiological noises. Accordingly, it returned separate estimates of the functional and noise components. We used the functional components for the changes in HbO as the final preprocessed measurements (due to the negative correlation with HbO, functional components for the changes in HbR were thus redundant after applying HMS, see Yamada et al. 2012).

As well as the preprocessing, channels with poor signal quality were detected despite the efforts to optimize optode contacts with the scalp. The scalp coupling index (SCI), which can effectively identify poor fNIRS signals in speech perception experiments (Pollonini et al. 2014; Mushtaq et al. 2019, 2021; Lawrence et al., 2021), was adopted. The signals with the two wavelengths were first bandpass filtered into 0.5–2.5 Hz that represents the cardiac elements captured by fNIRS and were correlated with each other. The higher correlation indicates better optode contacts. Following the criteria used in previous speech perception studies using fNIRS (Mushtaq et al. 2019, 2021; Lawrence et al., 2021), the worst 5% of channels (across all participants and sessions) were excluded for subsequent analyses. This threshold was set to ensure as many channels as possible (i.e., 95% of all channels) were preserved for statistical analyses (Mushtaq et al. 2019, 2021; Lawrence et al., 2021) especially when relative low number of participants (i.e., 10) were recruited in the current study. Individual numbers of bad channels and bad channel IDs are shown in TableS1 (*Supplementary Materials*).

#### Data Processing of Functional Activations and Connectivity

The preprocessed fNIRS activations were analysed to measure: (1) functional activation levels and (2) functional connectivity during both auditory and visual tests. We examined activation levels using block averaging across channels within several regions of interest (ROIs). This approach was employed because test-retest reliability in previous studies have shown that fNIRS activations are more reliably estimated when signals are averaged across small number of channels within a given ROI compared to when signals are analysed on a single-channel basis (Plichta et al. 2006; Schecklmann et al. 2008; Blasi et al. 2014; Wiggins et al. 2016). For the auditory tests, we focused on four ROIs of the bilateral auditory cortices (left: Channels 2, 3 and 7; right: Channels 24, 25 and 29), left inferior parietal lobule (Channels 11, 15, 16 and 20) and left frontal/prefrontal cortex (Channels 13, 17, 18 and 22). For the visual tests, we focused on two ROIs of the bilateral auditory cortices. Auditory cortices were chosen as we wanted to assess the functional neuroplasticity in auditory sensitivity in the auditory test and cross-modal maladaptation in the visual test in sensory auditory areas. The other two ROIs (left inferior parietal lobule and frontal/prefrontal cortex) were chosen for the auditory test since they reflect higher-order speech and language processing dominant in the left hemisphere (Hickok and Poeppel 2007). The left inferior parietal lobule is specifically associated with speech-in-noise perception (Alain et al. 2018) as well as semantic processing (Coslett and Schwartz 2018), whilst left frontal/prefrontal cortex is associated with listening effort (Wild et al. 2012; Wijayasiri et al. 2017; Rovetti et al. 2019; Sherafati et al. 2022). The fNIRS waveforms were temporally averaged across channels within each given ROI for each trial. The averaged waveform was then baseline-corrected by subtracting the mean of the 10-second pre-stimulus period and normalized by dividing the pre-stimulus’ standard deviation (Balconi et al. 2015; Balconi and Vanutelli 2016, 2017; Mutlu et al. 2020; Yorgancigil et al. 2022). The waveforms were then averaged across trials for each condition in each session. Because the haemodynamic responses peak at ~ 5 s after the stimulus presentation, the response amplitude for a given condition was measured as the mean amplitude across the 5–25 seconds’ period (according to the 20 s’ block duration) after stimulus onset.

Functional connectivity was quantified following the approach developed by Rissman et al. (2004) which measures correlations of beta-weight series across individual blocks (obtained via General Linear Model (GLM)) between different channels. Specifically, design matrices were first created for the auditory and visual tests in the three sessions (T0, T1 and T2), respectively. In each matrix, a boxcar regressor was created for every single block. The resting state was not included as a regressor based on the assumption that it did not actively trigger the haemodynamic responses and its activation level approximated to the global intercept. The canonical haemodynamic response function (HRF) was then convolved with the design matrix and the corresponding fNIRS signals were fitted using the convolved matrix via GLM (using the SPM-fNIRS toolbox) to obtain channel-wise beta weights. As such, a beta weight was obtained for every single block that reflected the level of activations of that block in each channel. This thus generated a beta-weight series for each condition (e.g., there were 12 blocks for the speech condition, hence giving a series of 12 beta values) for each channel. Pearson correlations of the beta-weight series were then calculated between individual channels (followed by Fisher-transform) as the values of connectivity between them. Such an approach has been successfully applied to quantify effective haemodynamic functional connectivity (Rissman et al. 2004; Ye et al. 2011; Gottlich et al., 2017; Antonucci et al. 2020; Pang et al. 2022).

### Statistical Analyses

Following acquirement of the behavioural (SRTs) and fNIRS data (activation levels and functional connectivity), statistically analyses were conducted to compare how these data changed between different testing sessions (T1 vs. T0, T2 vs. T0 and T2 vs. T1). Due to the relatively small number of participants, we applied bootstrapping instead of ANOVAs or T-tests to avoid requirement for assumptions of specific data distributions (e.g., normality). Specifically, data were resampled with replacement in each replication and a bootstrap distribution was obtained after 10,000 replications. The confidence intervals were measured using the bias-corrected and accelerated (BCa) approach (using the Matlab function ‘bootci’) which corrected the confidence limits by accounting for deviations of the bootstrapped mean from the sample mean and skewness of the distributions (Efron 1987; Efron and Tibshirani 1994). An effect was considered as statistically significant if the value of zero fell outside the [1–α] (α as the significance level set at 0.05) confidence interval of a given distribution. For the SRTs and fNIRS activation levels in each ROI, α was set at 0.05/3 to correct for the number of sessions (i.e., 3). For the functional connectivity, α was set at 0.05/(946*3) to correct for the total number of connectivity between all 44 channels (i.e., 946) and the number of sessions (i.e., 3).

## Results

### Behavioural Results

**Fig. 4:**
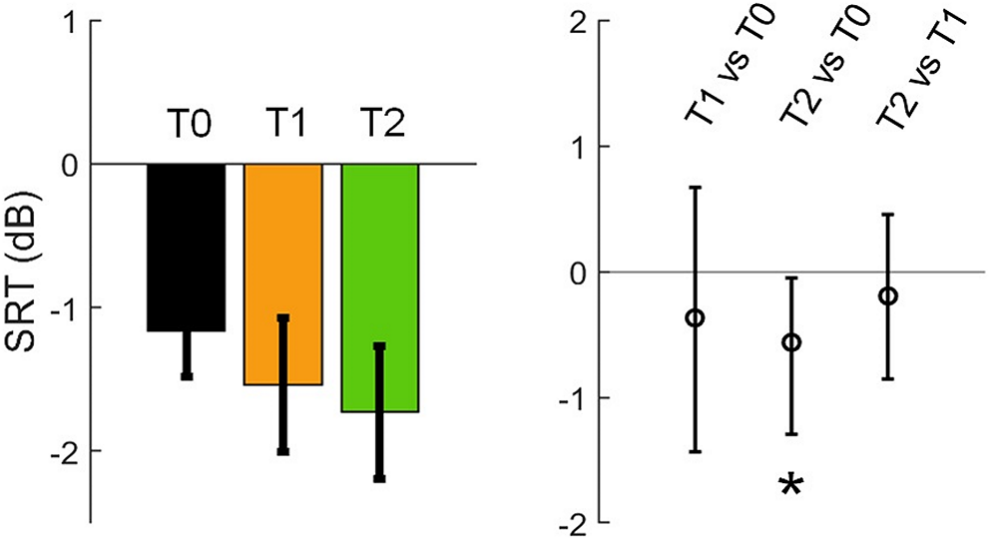
Speech-in-noise performances (SRT; lower SRT reflects better performance) across sessions. *Left panel*: SRTs at T0, T1 and T2. Error bars indicate standard errors of the means. *Right panel*: changes across sessions (T1 vs. T0, T2 vs. T0 and T2 vs. T1) with mean values indicated by circles in the middle and error bars indicating 95% confidence intervals (significance level α corrected at 0.05/3). The asterisk indicates statistical significance where zero is outside the confidence interval

Behavioural speech-in-noise performances were measured as SRTs. We found significantly lower SRT (i.e., better speech-in-noise performance) at T2 than at T0, but no significant differences between T1 and T0 or between T2 and T1 (Fig. 4; Table 1). This thus shows that speech-in-noise performance improved after retention (T2) but not immediately after training (T1).

**Table 1:** Statistical summary for the changes in SRT across sessions (T1 vs. T0, T2 vs. T0 and T2 vs. T1). Numbers in the brackets illustrate the 95% confidence intervals (significance level α corrected at 0.05/3) with corresponding effect sizes (Cohen’s d). The asterisk indicates statistical significance where zero is outside the confidence interval (in bold)

SRT (dB)		T1 vs. T0	T2 vs. T0	T2 vs. T1
	Confidence interval (CI) Effect size	[-1.455, 0.670] -0.248	**[-1.286**, -0.048]* -0.649*	[-0.877, 0.427] 0.212

### Neural Results

#### Auditory Tests

Functional activation levels and connectivity in response to auditory stimuli were compared between the three sessions. We conducted the comparisons separately for the speech and non-speech conditions, as well as for speech vs. non-speech.

**Fig. 5:**
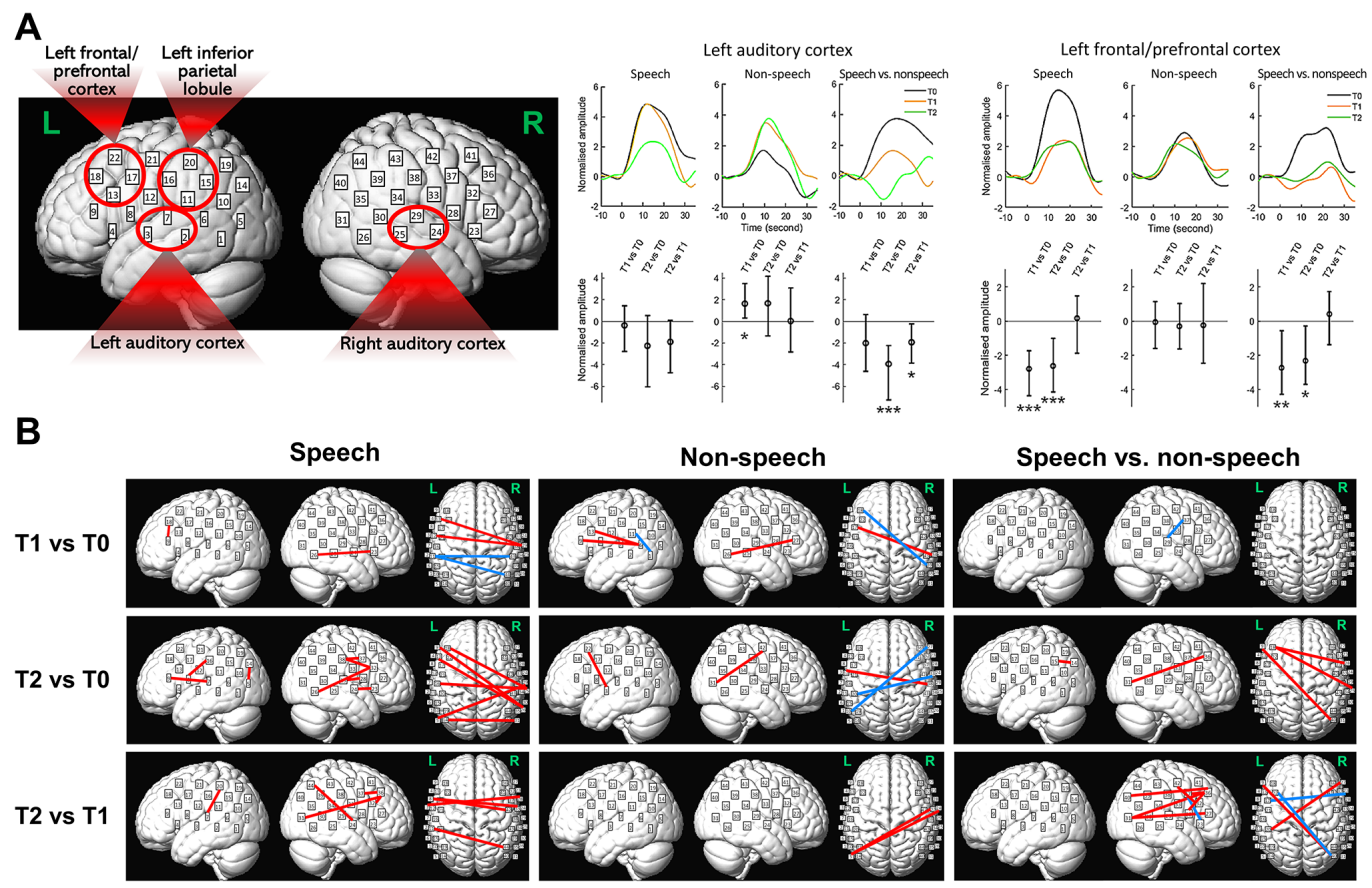
Changes in functional activation levels and connectivity during the auditory test across sessions (T1 vs. T0, T2 vs. T0 and T2 vs. T1) for the speech, non-speech and speech vs. non-speech conditions. **(A)** *Left*: ROIs for calculating functional activation levels indicated by red circles. ROIs include the bilateral auditory cortices (left: Channels 2, 3 and 7; right: Channels 24, 25 and 29), left inferior parietal lobule (Channels 11, 15, 16 and 20) and left frontal/prefrontal cortices (Channels 13, 17, 18 and 22). *Right*: changes in response amplitude in the ROI of the left auditory cortex and left frontal/prefrontal cortex which showed significant changes (no significant changes between sessions were found for the right auditory cortex and left parietal lobule, hence patterns for these two ROIs are not shown here). The left auditory cortex had significant increases in amplitudes in T1 vs. T0 for non-speech and decreases after retention (T2 vs. T0 and T2 vs. T1). The left frontal/prefrontal cortex had significant decreases in amplitude after training for speech and speech vs. non-speech (T1 vs. T0 and T2 vs. T0). Averaged normalised amplitudes for all three sessions (upper panels) and changes across sessions with mean values indicated by circles in the middle and the error bars indicating 95% confidence intervals (significance level α corrected at 0.05/3, lower panels). Single, double, and triple asterisks indicate statistical significance where zeros are outside the 95%, 99% and 99.9% confidence intervals, respectively. **(B)** Changes in functional connectivity. In each panel, significant changes (α corrected at 0.05/(964*3)) in intra- (within left and right hemispheres respectively) and inter-hemispheric connectivity are shown respectively (from left to right). The red and blue lines indicate the enhancement/increases and decreases in connectivity, respectively, showing that major enhancement occurred for speech after retention (T2 vs. T0 and T2 vs. T1)

For the activation levels, we found significantly changes in response magnitudes in the ROIs of left auditory cortex and left frontal/prefrontal cortex (see Fig. 5A; Table 2). Specifically, response amplitudes were significantly increased at post-training (T1) than the baseline (T0) in the left auditory cortex for the non-speech condition and significant decreases in responses after retention (T2 vs. T0 and T2 vs. T1) for speech than non-speech. In the left frontal/prefrontal cortex, responses amplitudes were significantly reduced at post-training than baseline (T1 vs. T0 and T2 vs. T0) in the left frontal/prefrontal cortex for the speech but not the non-speech condition. In addition, such decreases were also significantly greater for speech than for non-speech. No significant differences were found between sessions in right auditory cortex or left inferior parietal lobule. Effect sizes (Cohen’s *d*) of all significant effects were large (> 0.8) or medium-to-large (0.6–0.8) (Cohen 1988; see Table 2), indicating robustness of the results with the current sample size. Individual results for the left auditory and frontal/prefrontal cortices (speech vs. non-speech) are illustrated in Figure S2 (see *Supplementary Materials*) showing decreased activations after training in majority of the participants compared to baseline (T0) in these two ROIs.

For the functional connectivity, we found significant enhancements of connectivity for both speech and non-speech at T1 and T2 compared to T0 (as well as several decreases, see Fig. 5B). Importantly, however, these enhancements were dominant in the speech condition after retention (i.e., T2). There were 14 pairs of channels for T2 vs. T0 and 9 pairs of channels for T2 vs. T1 for the speech condition as opposed to no more than 4 pairs of channels in any other comparison for speech/non-speech where significant enhancements were found. These enhancements include intra- and inter-hemispheric connectivity between auditory (channels 2, 3, 7, 23, 24 and 29) and non-auditory (parietal and frontal) channels. For speech vs. non-speech, significant enhancements were found between non-auditory channels (posterior temporal lobe, parietal and frontal lobes) for T2 vs. T0 and T2 vs. T1 (Fig. 5B). These changes in functional connectivity thus corresponded to the behavioural changes where speech-in-noise performances improved after retention (T2) but not immediately after training (T1).

**Table 2:** Statistical summary for the changes in the activation levels across sessions (T1 vs. T0, T2 vs. T0 and T2 vs. T1) in specific regions of interest (ROIs) (left and right auditory cortices, left inferior parietal lobule, and left frontal/prefrontal cortex) during the auditory test. Numbers in the brackets illustrate the 95% confidence intervals (α corrected at 0.05/3) along with corresponding effect sizes. Single, double and triple asterisks indicate statistical significance where zeros are outside the 95%, 99% and 99.9% confidence intervals, respectively (in bold)

Task	ROI	Condition	Confidence interval (CI) and effect size	T1 vs. T0	T2 vs. T0	T2 vs. T1
Auditory task	Left auditory cortex	Speech	CI Effect size	[-2.846, 1.416] -0.035	[-5.848, 0.463] -0.455	[-4.919, 0.006] -0.618
		Non-speech	CI Effect size	**[0.284**, 3.501]* 0.708*	[-1.558, 4.231] 0.364	[-2.825, 3.000] -0.062
		Speech vs. non-speech	CI Effect size	[-4.590, 0.602] -0.490	**[-7.723**, -2.235]*** -0.970***	**[-3.876**, -0.217]* -0.682*
	Right auditory cortex	Speech	CI Effect size	[-2.421, 2.318] 0.003	[-2.844, 1.064] -0.245	[-4.992, 1.467] -0.159
		Non-speech	CI Effect size	[-1.581, 3.462] 0.202	[-1.635, 2.562] 0.124	[-3.927, 2.835] -0.073
		Speech vs. non-speech	CI Effect size	[-2.417, 1.811] -0.249	[-2.985, 1.242] -0.353	[-3.108, 2.186] -0.087
	Left inferior parietal lobule	Speech	CI Effect size	[-3.296, 2.200] -0.226	[-5.494, 1.639] -0.350	[-3.182, 1.944] -0.273
		Non-speech	CI Effect size	[-1.782, 0.407] -0.008	[-2.153, 1.631] -0.090	[-1.236, 1.591] -0.129
		Speech vs. non-speech	CI Effect size	[-2.496, 2.823] -0.274	[-4.357, 1.580] -0.387	[-3.221, 2.554] -0.144
	Left frontal/ prefrontal cortex	Speech	CI Effect size	**[-4.393**, -1.766]*** -1.062***	**[-4.179**, -1.058]*** -0.880***	[-1.969, 1.473] 0.027
		Non-speech	CI Effect size	[-1.705, 1.146] -0.056	[-1.593, 1.038] -0.158	[-2.481, 2.242] -0.124
		Speech vs. non-speech	CI Effect size	**[-4.261**, -0.529]** -0.904**	**[-3.732**, -0.370]* -0.761*	[-1.361, 1.709] 0.232

#### Visual Tests

Same as the auditory test, brain activation levels (response amplitudes in ROIs) and functional connectivity for the visual tests were compared between sessions. For the activation levels, we did not find any significant differences in beta-weights in any channel or response amplitudes in either ROI (the left or right auditory cortex) between sessions (see Table 3).

**Table 3:** Statistical summary for the changes in the activation levels across sessions (T1 vs. T0, T2 vs. T0 and T2 vs. T1) in specific ROIs (left and right auditory cortices) during the visual test. Numbers in the brackets illustrate the 95% confidence intervals (α corrected at 0.05/3) along with corresponding effect sizes

Task	ROI	Confidence interval (CI) and effect size	T1 vs. T0	T2 vs. T0	T2 vs. T1
Visual task	Left auditory cortex	CI Effect size	[-4.604, 0.453] -0.530	[-4.687, 1.180] -0.242	[-1.364, 4.269] 0.243
	Right auditory cortex	CI Effect size	[-1.736, 1.793] 0.140	[-2.238, 1.722] 0.098	[-2.615, 1.825] -0.027

**Fig. 6:**
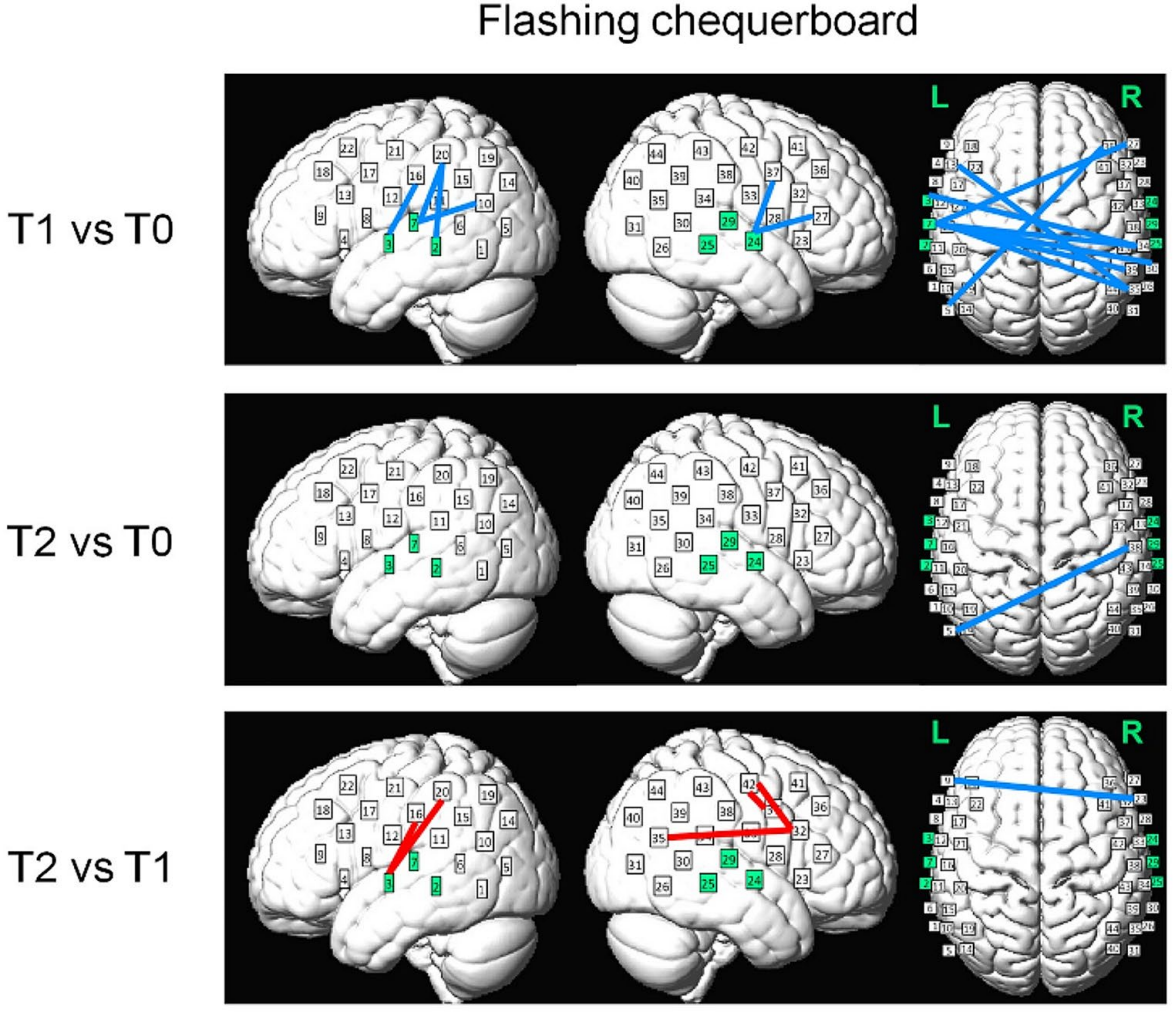
Changes in functional connectivity during the visual test across sessions (T1 vs. T0, T2 vs. T0 and T2 vs. T1). In each panel, significant changes (α corrected at 0.05/(964*3)) in intra- and inter-hemispheric connectivity are shown respectively (from left to right). The red and blue lines indicate the enhancement/increases and decreases in connectivity, respectively. Major changes were decreased connectivity between auditory and non-auditory cortices immediately after training (T1 vs. T0). Channels on the left and right auditory cortices (Channels 2, 3, 7, 24, 25 and 29) are highlighted in green

For the functional connectivity, changes were mainly found in T1 where significant decreases in connectivity were found between 14 pairs of channels for T1 vs. T0, where only one pair was found for T2 vs. T0 (see Fig. 6). Out of these 14 pairs for T1 vs. T0, only two pairs were those unrelated to auditory cortices (connectivity between channels 13 and 35 and between 5 and 36); the other 12 pairs were all between auditory cortices (10 pairs at channels 2, 3 and 7 on the left and 2 pairs at channel 24 on the right) and non-auditory regions in the parietal and frontal areas and temporo-parietal junctions. Therefore, the results show that brain connectivity between auditory cortices (especially the left auditory cortex) and higher-level non-auditory regions in response to the visual stimuli were significantly decreased immediately after training, but then such decreases vanished after retention.

## Discussion

### Neuroplasticity for Speech-in-Noise Processing in Older Adults Detected by fNIRS

Functional neuroimaging techniques, such as fMRI and PET, often face limitations in auditory research. For example, loud scanning noise in fMRI requires careful design of paradigms in auditory experiments (Hall et al. 1999, 2009; Blackman and Hall 2011; Peelle 2014) which could be tricky for hearing-impaired participants. PET, is invasive requiring injection of radioactive isotopes, hence limiting its feasibility of repetitive use for longitudinal studies (Saliba et al. 2016; Basura et al. 2018; Harrison et al. 2021). fNIRS, on the other hand, is non-invasive, acoustically silent and feasibly used longitudinally. In the current study, we used fNIRS to conduct a longitudinal study to examine auditory neuroplasticity in older adults. To our knowledge, this is the first study using fNIRS to examine neuroplasticity in terms of speech-in-noise perception. Most of our older adults (eight out of ten) had mild-to-moderate hearing loss, especially at high-frequencies (> 2 kHz), consistent with the real-life patterns of sensorineural hearing loss during normal ageing (Gopinath et al. 2009; Humes et al. 2010). Older adults often face challenges in listening to speech under noisy environments (Humes 1996), especially for those who have hearing loss (Souza and Turner 1994; Barrenäs and Wikström 2000; Humes 2008) and speech-based training has been provided aiming to improve their speech-in-noise perception (Stropahl et al. 2020; Bieber and Gordon-Salant 2021). Our results showed both behavioural and neural changes after training.

We showed significant behavioural improvements (i.e., speech-in-noise performances) after the retention period (T2), but not immediately after training (T1) compared to the pre-training baseline (T0) (Fig. 4). This corresponded to enhancements in functional connectivity during the auditory tests. Significant enhancements in connectivity were predominantly observed for the speech condition at T2 (T2 vs. T0 and T2 vs. T1), but not T1 (T1 vs. T0) (Fig. 5B). Such enhancements include greater intra- and inter-hemispheric connectivity between channels across temporal, parietal and frontal regions. This may indicate that changes in wide-spread functional connectivity could be potential indices for behavioural changes in speech-in-noise perception. This is also consistent with arguments that speech perception involves functioning of large-scale neural networks encompassing multiple wide-spread cortical regions that wire together rather than functioning of a single hub (Hickok and Poeppel 2007). As indicated in our results, such networks whose enhancements were observed include not only lower-order auditory/temporal regions, but also higher-order non-auditory (parietal and frontal) regions. It has been reported that parietal cortices are involved with short-term phonological storage (Buchsbaum and D’Esposito 2009), sensorimotor speech integration (Alho et al. 2014; Skipper et al. 2017) and semantic processing (Coslett and Schwartz 2018), whilst frontal cortices are related to effortful listening (Wild et al. 2012; Wijayasiri et al. 2017), phonological working memory maintenance (Strand et al. 2008; Liebenthal et al. 2013) and syntactic processing (Grodzinsky et al. 2021) during speech perception. Such higher-level functional connectivity is also reflective of the possible underlying structural connectivity. For example, studies using techniques like diffusion tensor imaging have shown strong anatomical neural connectivity between auditory and higher-level parietal and frontal cortices (e.g., Saur et al. 2008, 2010). Such connectivity includes ‘dorsal’ (temporal-frontal connectivity through parietal and premotor cortices) and ‘ventral’ (temporal-frontal connectivity through routes from the middle temporal lobe to ventrolateral prefrontal cortex) pathways (Hickok and Poeppel 2007; Saur et al. 2008, 2010; Rauschecker 2012). These pathways are potentially related to different speech and language processes as mentioned above, e.g., phonological and sensorimotor speech processing via the dorsal pathway and semantic and syntactic processing via the ventral pathway (Hickok and Poeppel 2007; Saur et al. 2008, 2010; Rauschecker 2012). Although the current functional connectivity measures cannot reveal which types of these processes may have been involved, we suggest that our results provide important initial findings to indicate the neuroplasticity of these functional networks for speech and language processing, especially in older adults. Furthermore, the enhancements of inter-hemispheric connectivity indicate the potential importance of coordination and cooperation between the two hemispheres for speech-in-noise perception, which is a result, to our knowledge, that has not been reported previously.

We also found neural changes in the ROI of the left auditory cortex in the auditory tests that correspond to the behavioural changes. Intriguingly, we found significant decreases in functional activations in the left auditory cortex comparing speech with non-speech at T2 (T2 vs. T0 and T2 vs. T1) (Fig. 5A). This is, however, *inconsistent* with our hypothesis predicting that auditory sensitivity, especially that to speech stimuli, should increase after training. A possible reason may be that the auditory stimuli consisted of not only target stimuli (speech/non-speech sentences), but also background noise (multi-talker babbles, see *Design* in *Methods and Materials*). It is plausible that auditory cortical activities responded to not only speech but also the background noise. While the current fNIRS measured neural responses using speech stimuli at individual SNR corresponding to 50% word correctness at T0, meaning that the intensity levels of background noise were substantial (average SNR was below zero, see *fNIRS experiments* in *Methods and Materials*). The decreased activations may thus be explained by suppression in neural responses to background noise in the left auditory cortex. It is noticeable that these significant decreases (speech vs. non-speech) were due to decreased responses in speech, while at the same time, increased responses to non-speech (see Fig. 5A and Table 2). We thus suggest that this can be interpreted as overall combined effects of neural suppression of background noise during speech listening (leading to decreased responses in the speech condition) and increases in general auditory sensitivity (leading to increased responses in the non-speech condition). This interpretation is consistent with previous studies showing that neural suppression of background noise could be more important than neural enhancement of target speech to achieve successful speech-in-noise perception in older adults with hearing loss (Petersen et al. 2017). Interestingly, these effects were significant only on the left, but not right, auditory cortex, further stressing the hemispheric specificity of speech processing (Hickok and Poeppel 2007). To our knowledge, this is the very first finding of possible background suppression observed by haemodynamic responses to speech in noise according to longitudinal changes.

Furthermore, we also observed neural changes can occur *before* the significant changes in behavioural performances. Specifically, functional activation decreased in the left frontal/prefrontal cortex during the auditory tests at both T1 and T2 compared to T0, hence taking place before the behavioural improvements that only emerged at T2. These decreases occurred for the speech condition and were significantly greater for speech than non-speech (see Fig. 5A; Table 2). This thus indicates that such effects were not merely driven by acoustics, but also higher-level speech-specific features like intelligibility. Previous research has demonstrated that activations in the left frontal/prefrontal regions reflect listening efforts during auditory and speech perception in populations with various hearing status, including young normal-hearing adults (Wild et al. 2012; Wijayasiri et al. 2017), older adults with normal hearing (Wong et al. 2009) and mild-to-moderate hearing loss (Rovetti et al. 2019), and cochlear implant listeners who have severe hearing impairment (Sherafati et al. 2022). Therefore, this result demonstrated reduced listening effort during speech-in-noise perception even *before* the occurrence of behavioural improvement and such reduction persisted after the retention period. This further indicates that this reduction at T1 could predict future behavioural outcomes in T2. However, we conducted some additional analyses but had not find significant correlations between changes in neural activations at any ROI or functional connectivity at T1 (vs. T0) and changes in SRT at T2 (vs. T0). This may be due to the relatively small sample size resulting in inadequate statistical powers. This may also be because reduced listening efforts reflect alleviation of participants’ mental burdens and exertions during speech-in-noise perception, an index that may be independent of speech recognition performances, so it may not be necessarily transferred into prediction of improvements in future behavioural outcomes.

We also observed significant decreases in functional connectivity between auditory cortices and non-auditory parietal and frontal regions during the visual (checkerboard flashing) test (T1 vs. T0), which also occurred *before* the significant behavioural changes. Previous studies have shown greater auditory cortical activities in hearing-impaired people when they process non-auditory (e.g., visual) stimuli possibly reflecting functional takeover of the auditory functions (Rouger et al. 2012; Campbell and Sharma 2014; Chen et al., 2016; Dewey and Hartley 2015; Corina et al. 2017) associated with worsened speech perception (Campbell and Sharma 2014). The current result may thus reflect decreases in cross-modal takeover after training. Also, this result should be the first time to indicate the possible takeover effects reflected by functional connectivity between auditory and higher-order speech-related areas. This may also reflect greater suppression of activities in auditory-related areas during visual stimulations. However, such decreases did not persist after retention and thus did not correspond to the changes in speech-in-noise performances. We argue that this may be because older participants in the current study had either normal hearing or mild-to-moderate hearing loss, while the takeover effects shown in the previous studies were reported in those with severe hearing loss (Campbell and Sharma 2014; Chen et al., 2016; Dewey and Hartley 2015; Corina et al. 2017). It is thus possible that, with less impaired hearing, our participants may have lower potentials for cross-modal neuroplastic changes. Therefore, while these decreases were observed immediately after training, they may be harder to persist, especially when the training had stopped during the retention period. Nonetheless, we demonstrated these longitudinal changes in cross-modal activations in healthy older participants that have not been reported in previous studies, hence illustrating the promises of using fNIRS to study such changes in more hearing-vulnerable populations in the future.

Taken together, our results demonstrated the auditory neuroplasticity using fNIRS where longitudinal changes in brain functions in response to auditory and visual stimuli occurred along with changes in behavioural (i.e., speech-in-noise) performances. Specifically, we found that large-scale functional connectivity in response to speech in noise was enhanced corresponding to the behavioural improvements. We also found corresponding decreases in left auditory cortical responses to speech vs. non-speech, possibly reflecting neural suppression of background noise that contributes the behavioural improvements. Crucially, we also demonstrated that neural changes, i.e., decreased left frontal/prefrontal responses to speech (reflecting reduced listening efforts) and decreased visual-elicited connectivity between auditory cortices and higher-order speech-related non-auditory areas (reflecting reduced cross-modal takeover and/or greater cross-modal suppression), occurred *before* the emergence of behavioural improvements. These changes can thus be seen as neural precursors that would not be detected solely through behavioural measurements, hence indicating predictive/prognostic potentials for treatments of speech-in-noise perception in hearing-impaired populations.

### Limitations and Future Research

The current finding that speech-in-noise performance was improved only after retention (T2) rather than immediately after training (T1) indicates that the training may have resulted in a longer/medium-term rather than an immediate behavioural effect. Alternatively, this may be due to learning effects of multiple experiment sessions. This would also apply to changes in neural activities observed here. Future studies including a control group without receipt of training would help to disentangle the training and learning effects. Nonetheless, an important goal of our study was to assess the promises of fNIRS to study auditory neuroplasticity alongside behavioural changes without much concerning about the exact driver of this plasticity. In this sense, it is less important to clarify the training and learning effects, whereas the speech-based training can be seen as a tool that helped facilitate the emergence of neuroplastic changes.

Second, another limitation was the small sample size, albeit the large or medium-to-large effect sizes of the significant results for the activation levels (see Table 2). This is due to the challenges of participant recruitment because the study required participants’ commitment to completing the entire 4-week home-based training process and multiple neuroimaging scanning sessions. More participants would be recruited to have greater statistical power in the future and to allow for better estimation of how neural changes are associated with behavioural changes. We suggest that the bootstrapping approach applied here has mitigated this potential concern for sample size, but a larger sample size would be needed to validate our results by future studies. Also, future research would apply fNIRS in those who have more severe hearing impairment and/or those with hearing protheses (e.g., hearing aids and cochlear implants) to further prove the promises of fNIRS in wider hearing-vulnerable populations.

Third, future work could improve reproducibility of fNIRS results. In more recent work of fNIRS, multiple short channels (comprised by optodes with distance as short as 8 mm) are used to detect systemic physiological confounds in the extracerebral layers (changes in arterial blood pressure, respiration, cardiac activities, etc.) that are not directly related to functional neural changes in the cortex (Tachtsidis and Scholkmann 2016). Subtracting these confounds detected by the short channels should give more robust and reliable neural signals. While the current study lacked short channels in the experimental setups, we applied bandpass filtering followed by appropriate methodologies (the HMS algorithm) to attenuate physiological confounds. While such approach has been proved to provide good test-retest reliability in fNIRS (see Wiggins et al. 2016), future work using short channels should provide further robustness to detect cortical neural responses, in particular, functional connectivity based on research using fMRI (Rissman et al. 2004) which has better signal-to-noise ratios than fNIRS. Furthermore, interindividual variability of channel locations may affect the reproducibility of current results. While accurate landmark digitalisations for every individual participant is difficult, the current study employed the standardised optode localisation approach without digitalisation. We have derived that, because of the high consistency of head sizes in our participants, the problem of channel locations may be minor (see the rationale discussed in *fNIRS Experiments* in *Methods and Materials*). However, this is still an unconfirmed estimation and reliability of the standardised approach to ensure channels largely locate at the same/similar anatomical brain regions across individuals is still not entirely clear. Future work could combine information of individual structural MRI that more accurately estimates which brain regions different channels correspond to and hence further attenuates biases due to individual differences in channel locations (Forbes et al. 2021).

Finally, cognitive assessments were not available for statistical analyses. Although there has not been evidence indicating that domain-general cognitive capacities (e.g., attention and working memory that are not specific to auditory speech perception) change according to speech-in-noise training, it is noteworthy that these capacities are associated with speech recognition performances in noise, especially in older adults (Schoof and Rosen 2014). It is valuable for future work to also look into how cognitive capacities may impact the *changes* in speech-in-noise recognition over time as well as changes in neural functions during speech-in-noise perception.

### Conclusion

To our knowledge, the current study is the first to use the optical neuroimaging technique of fNIRS to test and observe longitudinal changes in auditory functions in older adults. fNIRS is a tool that has unique advantages to assess and monitor functional brain activities in hearing-vulnerable populations over other functional neuroimaging techniques like fMRI and PET. Here, we demonstrated evidence for detecting neuroplasticity for speech-in-noise perception using fNIRS. Novel findings of functional neural changes were illustrated along with behavioural changes in longitudinal experiments in older adults after speech-in-noise training. Corresponding to improvements in speech-in-noise performances, we observed increased functional connectivity across wide-spread speech- and language-related regions reflecting enhancement of inter-regional coordination/cooperation to process speech, as well as decreased left auditory cortical responses to speech in noise possibly reflecting neural suppression of background noise. More interestingly, neural changes not only occurred at the same time with behavioural improvements, but also emerged as neural precursors *before* these improvements took place. Specifically, listening effort to speech and cross-modal takeover was reduced (decreased left prefrontal/frontal cortical activations during speech-in-noise listening and decreased connectivity between auditory and higher-order non-auditory areas during exposure of visual stimuli, respectively) before speech-in-noise performances could be improved. To our knowledge, these novel findings have not been previously reported. The findings thus open up new opportunities for future studies to base on to further investigate neuro-markers for functional changes during speech processing in older adults. We also argue that the current study should lay the ground for evaluating auditory neuroplasticity, as well as illustrate the promises of using fNIRS to detect such neuroplasticity, in wider hearing-impaired populations in the future, such as those who wear hearing protheses (e.g., hearing-aid and cochlear implant listeners).

## Electronic Supplementary Material

Below is the link to the electronic supplementary material.

Supplementary Material 1

## Data Availability

Data will be available upon request.
