# Supplementary material for: Neuroplasticity of Speech-in-Noise Processing in Older Adults Assessed by Functional Near-Infrared Spectroscopy (fNIRS)
Source: Brain Topogr. 2024 Jul 23;37(6):1139–57. doi: 10.1007/s10548-024-01070-2 (PMC11408581; doi:10.1007/s10548-024-01070-2)
Supplement: Supplementary file 1 — Supplementary Material 1 [file 10548_2024_1070_MOESM1_ESM.docx]

**Neuroplasticity of speech-in-noise processing in older adults assessed by functional near-infrared spectroscopy (fNIRS)**

Guangting Mai, Zhizhao Jiang, Xinran Wang, Ilias Tachtsidis, Peter Howell

**S1. Individual bad channels**

Bad channels were detected using scalp coupling index (SCI), which can effectively identify poor fNIRS signals in speech perception experiments (see main text for details). Channels with the lowest 5% SCI across all participants and test sessions (SCI was averaged across the auditory and visual tasks for each session) were detected as bad channels and were excluded for subsequent analyses. **Table S1** shows the numbers of bad channels and the bad channel IDs for all individual participants at all testing sessions (T0, T1 and T2). As a reference, we include **Figure S1**, which shows the anatomical positions of all 44 channels and the relevant ROIs, so that readers can conveniently find out the corresponding positions of these bad channels.

**
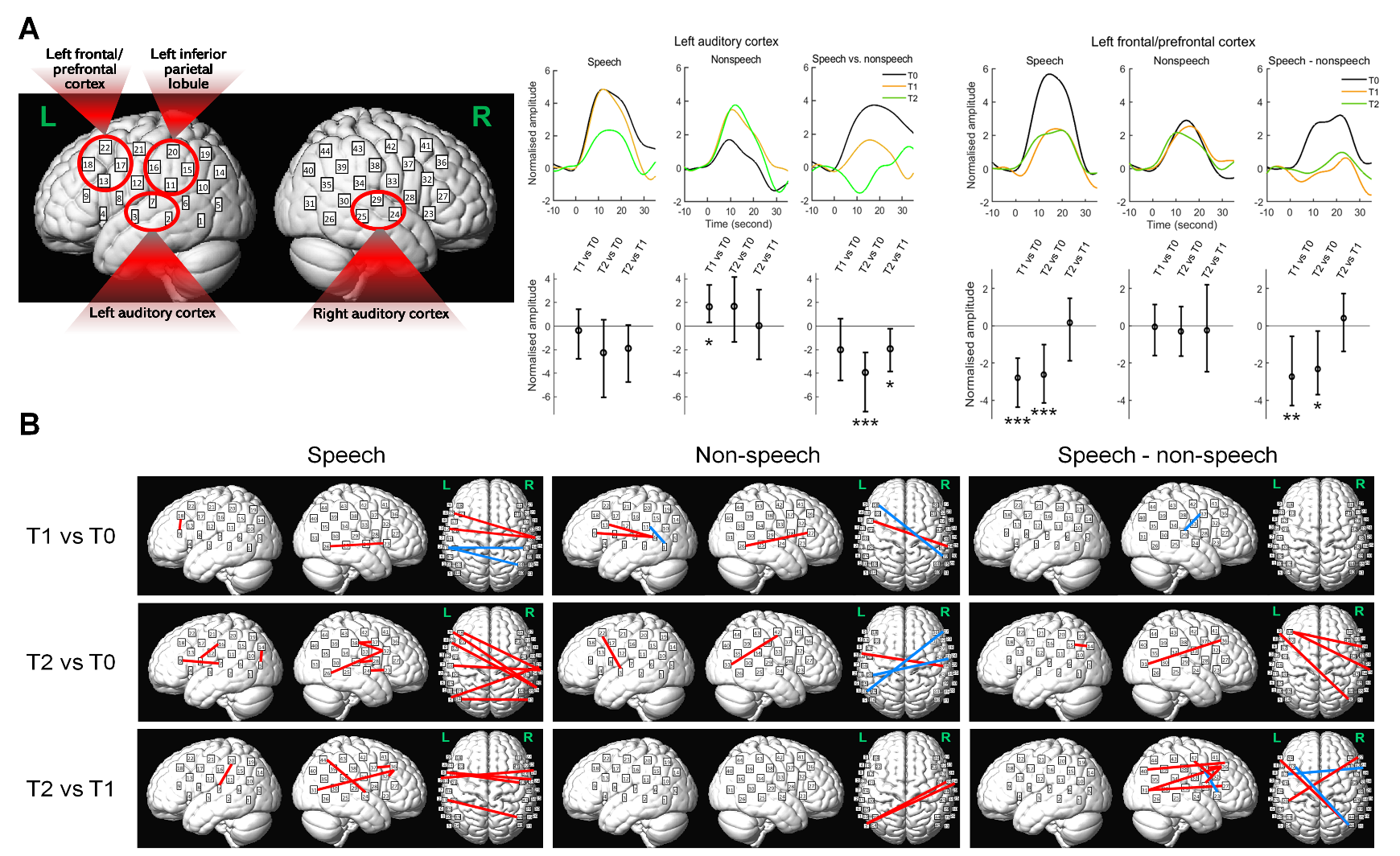
**

**Figure S1.** Anatomical positions of channels and relevant ROIs. This figure is the same as the left panel of **Figure 5A** in the main text.

**Table S1.** Numbers of bad channels and bad channel IDs for all 10 individual participants.

|  | T0 | | T1 | | T2 | |
| --- | --- | --- | --- | --- | --- | --- |
|  | Number of bad channels | Bad channel IDs | Number of bad channels | Bad channel IDs | Number of bad channels | Bad channel IDs |
| Participant 1 | 9 | 11, 16, 21, 22, 31, 35, 40, 41, 42 | 6 | 10, 16, 18, 20, 21, 42 | 9 | 16, 20, 21, 22, 26, 35, 41, 42, 43 |
| Participant 2 | 0 | **-** | 1 | 2 | 0 | - |
| Participant 3 | 3 | 10, 16, 35 | 3 | 16, 21, 42 | 3 | 10, 14, 38 |
| Participant 4 | 0 | - | 0 | - | 0 | - |
| Participant 5 | 0 | - | 0 | - | 1 | 2 |
| Participant 6 | 4 | 7, 19, 40, 42 | 9 | 5, 10, 14, 15, 19, 21, 35, 42, 43 | 9 | 10, 14, 15, 20, 30, 34, 35, 39, 42 |
| Participant 7 | 0 | - | 2 | 30, 35 | 1 | 39 |
| Participant 8 | 1 | 5 | 1 | 43 | 1 | 39 |
| Participant 9 | 0 | - | 0 | - | 0 | - |
| Participant 10 | 0 | - | 0 | - | 0 | - |

**S2. Individual results for functional activation levels in the left auditory cortex and left frontal/prefrontal cortex (speech vs. non-speech)**

**
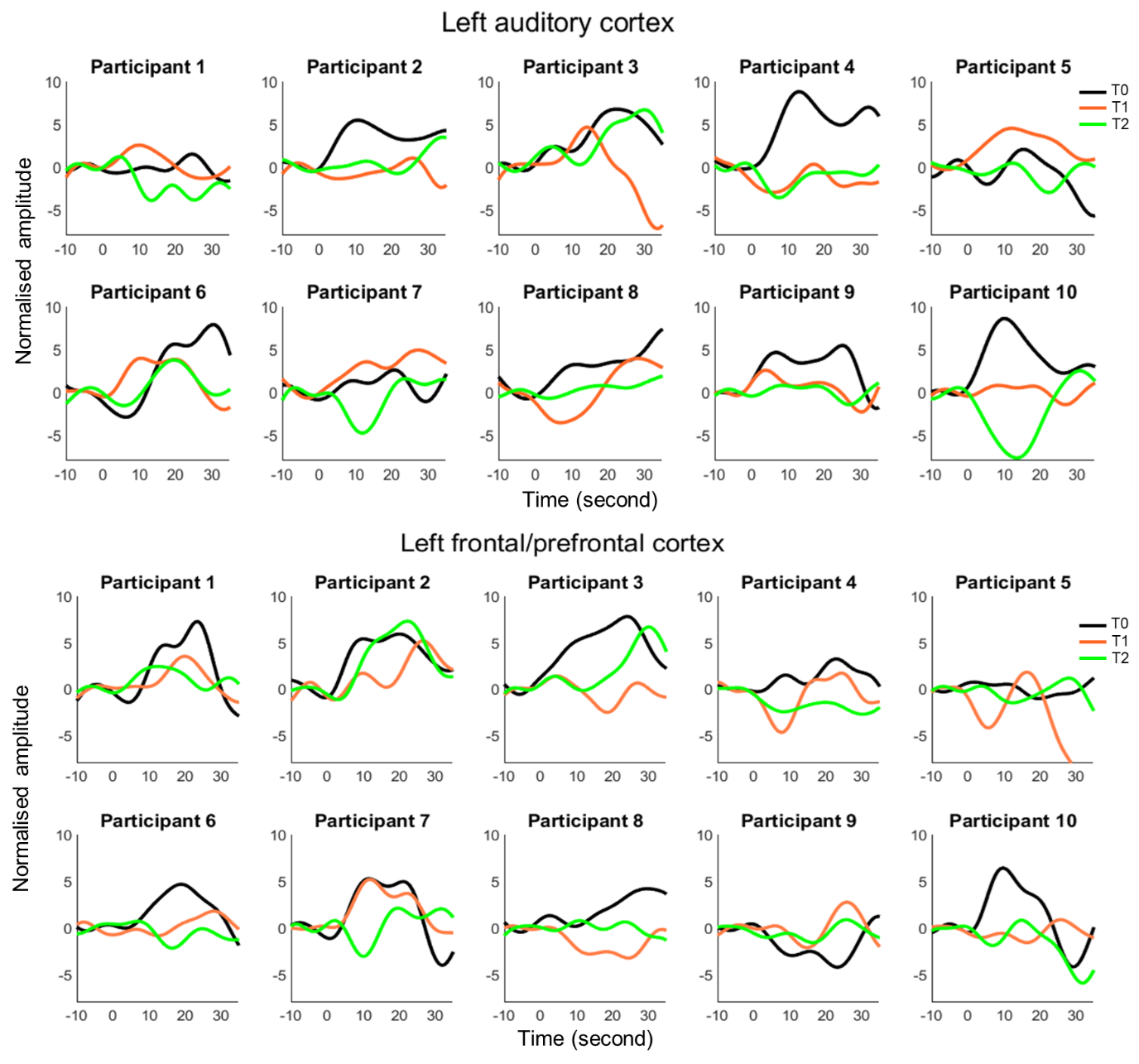
Figure S2.** Individual results (Participant 1 to 10) for functional activation levels during the auditory test across sessions (T0, T1 and T2).

**Figure S2** illustrates individual results for ROIs which showed statistical significance (the left auditory cortex and left frontal/prefrontal cortex) for speech vs. non-speech. The results show that majority of the participants’ activation levels decreased after training (T1 and T2) compared to baseline (T0). Statistical significances are shown in **Figure 5A** and **Table 2**.
